# Clinical profile and pattern of second primary tumors in HPV-positive and HPV-negative oropharyngeal cancer

**DOI:** 10.1590/1980-549720260046

**Published:** 2026-08-17

**Authors:** Matheus de Abreu, Dandara Menezes de Araujo Oliveira, Janaina Naiara Germano, Luiz Paulo Kowalski, Maria Paula Curado

**Affiliations:** I A.C. Camargo Cancer Center, International Research Center, Department of Epidemiology and Statistics Nucleus – São Paulo (SP), Brazil.; II A.C. Camargo Cancer Center, Department of Head and Neck Surgery and Otorhinolaryngology Department – São Paulo (SP), Brazil.

**Keywords:** Oropharyngeal neoplasms, Neoplasms, Second Primary, HPV, Alcohol Drinking, Tobacco Smoking, Neoplasias Orofaríngeas, Segunda Neoplasia Primária, HPV, Consumo de Bebidas Alcoólicas, Hábito Tabágico

## Abstract

**Objectives:**

To evaluate the clinical-epidemiological profile of patients with oropharyngeal cancer (OPC) positive for human papillomavirus (HPV+) and negative for human papillomavirus (HPV−), and to compare differences in the development of second primary tumors (SPT).

**Methods:**

Retrospective cohort with patients diagnosed with OPC. Sociodemographic/clinical data, p16-status, alcohol/tobacco consumption were extracted. Absolute/relative frequencies, odds ratios (OR), prevalence of HPV and SPT were calculated.

**Results:**

Of 538 patients with OPC, 59.8% (n=322) were HPV+; 40.2% (n=216) HPV−. The prevalence of SPTs was higher among HPV− (21.8%; n=47) than HPV+ (13.0%; n=42). Non-smokers were 4.5 times more likely to have HPV+ OPC (OR 4.58; confidence interval — CI 2.48–8.67), and non-drinkers were 2.06 times more likely (OR=2.06; CI 1.15–3.72). Nodal involvement was associated with a 2.77-fold higher likelihood of HPV+ (OR 2.77; CI 1.70–4.56); lymphoepithelial topographies with a 2.13-fold higher likelihood (OR 2.13; CI 1.37–3.31). SPTs occurred most in the skin (17.3%, n=9) and penis/prostate (15.4%, n=8) in HPV+, whereas in HPV- in head and neck (36.2%, n=22) and respiratory tract (18.0%, n=11). The interval between OPC diagnosis and SPT diagnosis was shorter in the HPV+ (median: 40 months).

**Conclusion:**

SPT pattern in OPC is not equal across HPV-groups. HPV- showed a higher prevalence of SPTs, more often involving the head and neck/respiratory tract, which may reflect the cumulative effect of tobacco/alcohol exposure. In HPV+, the lower SPT prevalence and different tumor distribution may indicate a more limited field cancerization effect. The shorter time to SPT diagnosis in HPV+ suggests that surveillance strategies may need to consider distinct risk profiles according to HPV-status.

## RESUMO

**Objetivos:**

Avaliar o perfil clínico-epidemiológico de pacientes com câncer de orofaringe (CO) positivo para papilomavírus humano (HPV+) e negativo para papilomavírus humano (HPV−) e comparar diferenças no desenvolvimento de segundos tumores primários (STP).

**Métodos:**

Coorte retrospectiva com pacientes diagnosticados com CO. Foram extraídos dados sociodemográficos/clínicos, status-p16, consumo de álcool/tabaco. Calcularam-se frequências absolutas/relativas, razões de chances (OR), prevalência de HPV/STP.

**Resultados:**

Dos 538 pacientes com CO, 59,8% (n=322) eram HPV+; 40,2% (n=216) HPV−. A prevalência de STP foi maior entre HPV− (21,8%; n=47); HPV+ (13,0%; n=42). Não fumantes apresentaram probabilidade 4,5 vezes maior de ter CO-HPV+ (OR 4,58; IC 2,48–8,67); não etilistas tiveram probabilidade 2,06 vezes maior (OR=2,06; IC 1,15–3,72). O envolvimento linfonodal associou-se a uma probabilidade 2,77 vezes maior de HPV+ (OR 2,77; intervalo de confiança — IC 1,70–4,56); middleografias linfoepiteliais, a uma probabilidade 2,13 vezes maior (OR 2,13; IC 1,37–3,31). Os STP ocorreram mais em pele (17,3%, n=9) e pênis/próstata (15,4%, n=8) em HPV+, enquanto HPV− em cabeça e pescoço (36,2%, n=22) e trato respiratório (18,0%, n=11). O intervalo entre o diagnóstico de CO-STP foi menor em HPV+ (mediana: 40 meses).

**Conclusão:**

O padrão de STP no CO difere entre os grupos HPV. O grupo HPV− apresentou maior prevalência de STP, frequentemente envolvendo cabeça/pescoço e trato respiratório, possivelmente refletindo o efeito cumulativo do tabaco/álcool. No HPV+, a menor prevalência e distribuição distinta dos tumores podem indicar um efeito mais limitado de cancerização de campo. O menor tempo até o diagnóstico de STP no HPV+ sugere que estratégias de vigilância devem considerar perfis de risco distintos conforme o status-HPV.

## INTRODUCTION

Globally, approximately 106,400 new cases and 52,305 deaths from oropharyngeal cancer (OPC) were estimated in 2022^1^. Human papillomavirus (HPV) infection is one of the risk factors, present in around 30% of cases worldwide^2^. While the incidence of head and neck cancer (HNC) (which includes oral cavity, oropharynx and larynx cancers) associated with tobacco consumption is declining, there has been an increase in oropharyngeal tumors related to HPV^3^. In England, it has been estimated that, in 20 years, the majority of HNC will be HPV-positive (HPV+)^4^. In developed countries, OPC associated with HPV represents 17 to 56% of neoplasms, while in less developed countries the incidence is approximately 13%^2,5^. In Brazil, 14.4% of OPC cases are attributable to HPV infection, considering both genders^6^. Data on the prevalence of HPV by sex in OPC in South America are scarce; the prevalence has been reported to range from 4.0 to 59.1%^7-11^.

Malignant neoplasms related to HPV present clinical and epidemiological aspects that differ from those associated with tobacco consumption^2^. The tonsils and base of the tongue are the most common sites affected by HPV-related oropharyngeal squamous cell carcinoma (OPSCC), which are typically diagnosed at less advanced T stages but are often associated with lymph node metastases^12^. Currently, HPV+ OPSCC is more commonly diagnosed in individuals over 50 years old^13^, with low or no tobacco consumption^8^, in countries with higher Human Development Index (HDI)^2^.

Persistent tobacco and alcohol consumption is an indicator of risk behavior for the development of second primary tumor (SPT)^14,15^. A systematic review revealed discrepant rates in the development of SPT in HNC, with an average incidence of 13.2% between studies^16^. The study by Leoncini and collaborators (2018)^17^ identified a prevalence of 7.7% of SPT^17^. The sites most affected by SPT in HNC were the lungs, head and neck region, and esophagus^16,17^.

Although the role of tobacco and alcohol in HNC carcinogenesis is well established^18^, limited evidence is available on differences in the clinical profile and the occurrence of SPTs according to HPV status in OPSCC. Evaluating the distribution and timing of SPTs in HPV+ and HPV-negative (HPV-) patients may improve understanding of distinct disease patterns and support more tailored surveillance strategies.

## METHODS

### Sample population

This retrospective cohort study included patients diagnosed with OPSCC at the A. C. Camargo Cancer Center (ACCCC). ACCCC is a tertiary cancer hospital located in São Paulo, Brazil, and a national referral center that treats both public and private patients (insured and self-paying), with a predominance of privately insured patients from different regions of the country. Cases with a primary diagnosis of OPSCC (morphology code 8070/3) were identified between 2000 and 2022. Cases were classified according to the International Classification of Diseases for Oncology, 3^rd^ edition (ICD-O-3), including the following topography codes: C01.9 (base of tongue), C05.1 (soft palate), C05.2 (uvula), C09.0 (tonsillar fossa), C09.1 (tonsillar pillar), C09.8 (overlapping lesion of tonsil), C09.9 (tonsil, NOS), C10.0 (vallecula), C10.1 (anterior surface of epiglottis), C10.2 (lateral wall of oropharynx), C10.3 (posterior wall of oropharynx), C10.8 (overlapping lesion of oropharynx), and C10.9 (oropharynx, NOS). Cases without available p16 immunohistochemistry (IHC) results were excluded from the analysis. The number and characteristics of excluded cases are presented in Supplementary Table 1.

### Study variables

The sociodemographic variables included sex (male and female); age, categorized into three groups (18–49, 50–69, and ≥70 years); self-reported skin color (White and non-White, including Black and Brown individuals); and education level (≤9, 10–12, and >12 years). Lifestyle variables included self-reported smoking and alcohol consumption, classified as never, current, or former smoker and/or drinker. Former smokers and former drinkers were defined as individuals who had stopped smoking or drinking for more than one year.

Clinical variables included tumor topography, grouped as lymphoepithelial (C01.9, C09.0, C09.1, C09.8, C09.9, C10.0) and non-lymphoepithelial (C05.1, C05.2, C10.1, C10.2, C10.3, C10.8, C10.9), clinical stage, tumor size (cT), nodal involvement (cN), distant metastasis (cM), p16 status, date of OPSCC diagnosis, presence of a second primary tumor identified at diagnosis or during follow-up, topography of the second primary tumor, and date of SPT diagnosis. Cases were staged according to the 8^th^ edition of the American Joint Committee on Cancer (AJCC).

### p16 IHQ

Information on p16 status was obtained from the anatomopathological reports of the ACCCC. IHC was performed on formalin-fixed, paraffin-embedded tumor tissue sections (4 μm) using the monoclonal mouse anti-human p16INK4a antibody (clone E6H4™, Ventana Medical Systems) and the OptiView DAB IHC detection kit (Ventana Medical Systems) on a BenchMark ULTRA automated staining platform (Ventana Medical Systems), according to the manufacturer's instructions. Cases showing strong and diffuse p16 expression in more than 70% of tumor cells were considered positive for HPV infection. The analyses were conducted by an operator blinded to the patients’ clinical information.

### Statistical Analysis

Descriptive statistics were conducted to outline the sociodemographic and clinical characteristics of all patients included in the study, stratified according to HPV status (HPV+ and HPV−). All variables were described using absolute and relative frequencies. The chi-square test was used to assess the association between HPV status (dependent variable) and the independent variables, with p<0.05 considered statistically significant. The prevalence of second primary tumors (SPT) was calculated according to HPV status. The mean and median time intervals between the diagnosis of the primary OPSCC and the SPT were calculated. All statistical analyses were performed using the R programming language (version 4.4.1) within the RStudio environment (version 2024.09.0+375).

Odds ratios (OR) with corresponding 95% confidence intervals (95%CI) were estimated using logistic regression analysis to evaluate factors associated with HPV+, status, defined according to p16 expression as the outcome variable. Covariates with p<0.20 in the simple logistic regression analysis, as well as variables considered clinically or epidemiologically relevant, were eligible for inclusion in the multiple logistic regression model. A backward stepwise approach was applied for model selection. The order of entry of the variables was defined according to the p-value from the simple model, from lowest to highest. In the multiple logistic regression analysis, only variables with less than 5% missing values were considered. The final model was defined based on the following criteria:

1. no change in OR>10%;
2. statistical significance of variables within the multiple model (p<0.05); and
3. the total degrees of freedom allowed for the outcome. The Hosmer-Lemeshow test was applied to assess model fit.

### Ethics statement

This study was approved by the Human Research Ethics Committee of ACCCC (Certificate of Presentation for Ethical Appreciation — CAAE: 80177317.9.0000.5432; #7.234.641), and was conducted in compliance with the tenets of the World Medical Association Declaration of Helsinki.

#### Data availability statement:

the data underlying this article cannot be publicly shared due to ethical and privacy restrictions.

## RESULTS

Of the 538 patients diagnosed with OPSCC between 2000 and 2022, 59.8% (n=322) were HPV+ and 40.2% (n=216) were HPV−. Among the HPV+ patients, 79.2% (n=255) were male, compared with 85.2% (n=184) among HPV− patients. The prevalence of HPV+ OPSCC was 58.1% among men and 67.7% among women. Among non-White individuals, HPV− cases (n=51; 27.3%) were more than twice as frequent as HPV+ cases (n=48; 17.3%). Regarding education, 67.6% (n=117) of HPV+ patients had more than 12 years, whereas among HPV− patients the most frequent was <10 years (n=59; 46.8%). HPV+ patients were more frequently non-smokers (n=140; 44.2%), whereas HPV− patients were smokers (n=121; 56.3%) and alcohol drinkers (n=109; 51.7%) (Table 1).

**Table 1:** Sociodemographic and clinical characteristics of oropharyngeal squamous cell carcinoma (OPSCC) patients stratified by human papillomavirus (HPV) status. Crude odds ratios (OR) comparing HPV-positive (HPV+) versus -negative (HPV−) tumors (simple logistic regression). A.C.Camargo Cancer Center (2000–2022).

		HPV+	HPV-	p-value	Odds Ratio (simple models)		
					OR	95%CI	p-value
**Gender**							
	Male	255 (79.2)	184 (85.2)	0.078	1.00		
	Female	67 (20.8)	32 (14.8)		1.51	(0.96 – 2.42)	0.079
**Age (years)**							
	<50	83 (25.8)	28 (13.0)	0.001	2.80	(1.50 – 5.30)	0.001
	50 – 69	202 (62.7)	153 (70.8)		1.25	(0.75 – 2.08)	0.390
	>69	37 (11.5)	35 (16.2)		1.00		
**Ethnicity**							
	White	229 (82.7)	136 (72.7)	0.010	1.79	(1.14 – 2.80)	0.010
	Non-White	48 (17.3)	51 (27.3)		1.00		
	Missing	45	29				
**Education (years)**							
	<10	13 (7.5)	59 (46.8)	<0.001	1.00		
	10 – 12	43 (24.9)	39 (31.0)		5.00	(2.44 – 10.81)	<0.001
	>12	117 (67.6)	28 (22.2)		18.96	(9.42 – 40.70)	<0.001
	Missing	149	90				
**Smoking**							
	Smoker	68 (21.5)	121 (56.3)	<0.001	1.00		
	Former smoker	109 (34.4)	67 (31.2)		2.89	(1.90 – 4.44)	<0.001
	Non-smoker	140 (44.2)	27 (12.6)		9.22	(5.62 – 15.57)	<0.001
	Missing	5	1				
**Alcohol consumption**							
	Drinker	142 (46.0)	109 (51.7)	<0.001	1.00		
	Former drinker	35 (11.3)	69 (32.7)		0.39	(0.24 – 0.62)	<0.001
	Non-drinker	132 (42.7)	33 (15.6)		3.07	(1.96 – 4.90)	<0.001
	Missing	13	5				
**Tumor middleography**							
	Lymphoepithelial	238 (73.9)	108 (50.0)	<0.001	2.83	(1.97 – 4.09)	<0.001
	Non-lymphoepithelial	84 (26.1)	108 (50.0)		1.00		
**Clinical staging**							
	I/II	207 (66.1)	15 (8.2)	<0.001	1.00		
	III	84 (26.7)	59 (32.2)		0.10	(0.05 – 0.18)	<0.001
	IV	23 (7.2)	109 (59.6)		0.02	(0.01 – 0.02)	<0.001
	Missing	8	33				
**cT (TNM)**							
	cT1/cT2	165 (53.7)	81 (39.9)	0.002	1.75	(1.22 – 2.51)	0.002
	cT3/cT4	142 (46.3)	122 (60.1)		1.00		
	Missing	15	13				
**cN (TNM)**							
	cN0	54 (17.3)	123 (59.4)	<0.001	1.00		
	cN+	259 (82.7)	84 (40.6)		3.27	(2.19 – 4.92)	<0.001
	Missing	9	9				
**cM (TNM)**							
	cM0	293 (92.7)	190 (90.5)	0.357	1.34	(0.71 – 2.51)	0.359
	cM1	23 (7.3)	20 (9.5)		1.00		
	Missing	6	6				
**Second primary tumor**							
	Yes	42 (13.0)	47 (21.8)	0.008	0.54	(0.34 – 0.86)	0.008
	No	280 (87.0)	169 (78.2)		1.00		

HPV: human papilomavírus; OR: odds ratios; 95%CI: 95% Confidence Interval; cT (TNM): extent/size of the primary tumor (clinical stage – TNM classification); cN (TNM): lymph node involvement (clinical stage – TNM classification); cM (TNM): distant metastasis (clinical stage – TNM classification).

Lymphoepithelial topographies were more common among HPV+ OPSCC patients (n=238; 73.9%). HPV+ patients were more frequently diagnosed at early clinical stages (I/II) (n=207; 66.1%), whereas HPV− patients were more diagnosed at stage IV (n=109; 59.6%). Regarding clinical tumor classification (cT), 53.7% (n=165) of HPV+ tumors were classified as T1/T2, compared with 39.9% (n=81) of HPV− tumors. Lymph node involvement (cN+) was observed in 82.7% (n=259) of HPV+ cases and in 40.6% (n=84) of HPV− cases. Distant metastasis occurred in fewer than 10% of patients in both groups (HPV+ 7.3%, n=23; HPV− 9.5%, n=20) (Table 1). SPT were identified in 89 (16.5%) patients. The prevalence of SPTs was higher among HPV− patients (21.8%; n=47) than among HPV+ patients (13.0%; n=42), corresponding to a prevalence ratio of 1.67 (95%CI 1.14–2.44) for HPV- compared with HPV+ (Table 1).

**Figure 1:**
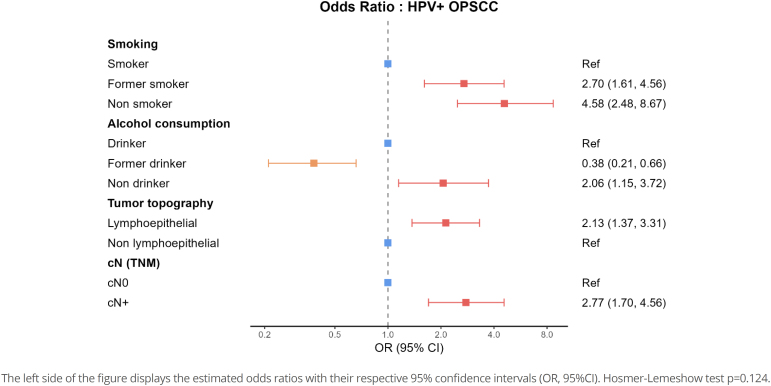
Adjusted logistic regression analysis (multiple model) of 538 patients with oropharyngeal squamous cell carcinoma (OPSCC), comparing human papillomavirus-positive (HPV+) versus -negative (HPV−) tumors. A.C.Camargo Cancer Center (2000–2022).

In the multivariable logistic regression model, smoking, alcohol consumption, tumor topography, and nodal involvement (cN) were independently associated with HPV status in OPSCC. Non-smokers were 4.5 times more likely to have HPV+ OPSCC (OR 4.58; 95%CI 2.48–8.67), and non-drinkers were 2.06 times more likely (OR=2.06; 95%CI 1.15–3.72). Clinically, nodal involvement (cN+) was associated with a 2.77-fold higher likelihood of HPV+ (OR 2.77; 95%CI 1.70–4.56) and lymphoepithelial topographies a 2.13-fold higher likelihood of HPV+ (OR 2.13; 95%CI 1.37–3.31) (Figure 1).

**Figure 2:**
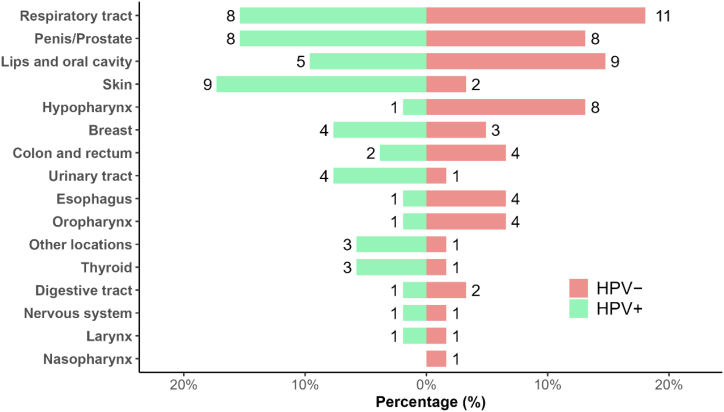
Topography of second primary tumors in patients with oropharyngeal squamous cell carcinoma (OPSCC) stratified by human papillomavirus (HPV); A.C.Camargo Cancer Center (2000–2022).

**Figure 3:**
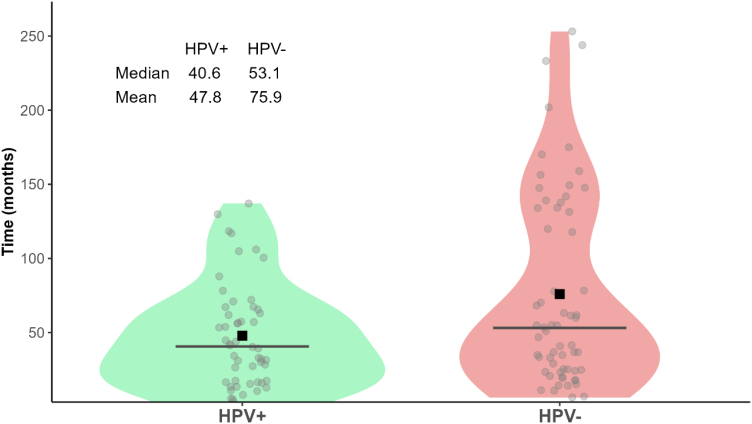
Interval between oropharyngeal squamous cell carcinoma (OPSCC) diagnosis and second primary tumor diagnosis (median and mean). A.C.Camargo Cancer Center (2000–2022).

Among HPV+ patients, SPTs occurred most frequently in the skin (17.3%, n=9), respiratory tract (15.4%, n=8), and penis/prostate (15.4%, n=8), whereas in HPV- they occurred in the pharynx (21.3%, n=13), respiratory tract (18.0%, n=11), and lips and oral cavity (14.8%, n=9) (Figure 2; Supplementary Table 2). The interval between OPSCC diagnosis and SPT diagnosis was shorter in the HPV+ group (median, 40.6 months; mean, 47.8 months) than in the HPV- (median, 53.1 months; mean, 75.9 months) (Figure 3).

## DISCUSSION

Our findings suggest that HPV status is related to differences in both the clinical profile of OPSCC patients and the occurrence of SPTs. HPV+ patients were less strongly associated to classical risk factors such as tobacco and alcohol, whereas HPV- patients showed a higher prevalence of SPTs, particularly in the head and neck and respiratory tract. In addition, the shorter interval between OPSCC and SPT diagnosis in HPV+ patients suggests that the timing of SPT occurrence may also differ according to HPV status.

### Clinical and sociodemographic profile of human papillomavirus-positive and -negative oropharyngeal squamous cell carcinoma patients

The proportion of HPV+ OPSCC has increased over time^7,9,11,19^. De Cicco et al. (2020)^8^ reported an HPV prevalence of 59.1% among patients diagnosed between 1984 and 2014^8^. In our study, which included patients diagnosed between 2000 and 2022, HPV prevalence was 59.8%, consistent with estimates reported in more recent cohorts. However, earlier studies using p16 IHC reported substantially lower HPV prevalence, such as 20.6^20^, 20.9^7^, and 17.7%^19^, possibly related to differences in study period, population characteristics, and study design.

A significant change in the age distribution of HPV+ OPSCC has been observed, with an exponential and continuous increase in incidence among older individuals^13^. In our study, the typical profile of HPV+ OPSCC patients included individuals aged 50 to 69 years. Similar findings have been reported in other studies, which have shown an increase in the average age of HPV+ OPSCC patients^13,21^, with the median age rising from 52 to 59 years^21^. These findings indicate a shift in the age profile of HPV+ OPSCC, with cases increasingly occurring among middle-aged and older adults rather than predominantly younger individuals. In addition, our findings indicate that HPV+ OPSCC was more common among individuals with higher educational attainment. Consistently, de Martel et al. (2017)^22^ reported that the proportion of OPSCC caused by HPV infection exceeds 40% in developed regions such as North America and Europe^22^, supporting a distinct epidemiological pattern compared with tobacco-related head and neck cancers, more often observed in populations with higher socioeconomic and educational levels.

In our cohort, HPV+ OPSCC patients showed lower exposure to classical risk factors, with more than 40% reporting no history of smoking. This finding is consistent with Gillison et al. (2008)^23^, who observed that p16-positive patients were more likely to have never smoked and had significantly lower tobacco exposure^23^. Similarly, Schostag et al. (2022)^24^ reported that only 20% of HPV+ patients had a history of smoking^24^. A similar pattern was observed for alcohol consumption, with HPV+ OPSCC patients being approximately twice as likely to be non-drinkers, consistent with findings reported by Gillison et al. (2015)^25^ and De Cicco et al. (2020)^8,25^.

Lymphoepithelial topographies were more strongly associated with HPV+ tumors than with HPV− tumors. A systematic review reported that OPSCC arising in the tonsils and base of tongue shows a higher prevalence of HPV infection, ranging from 56 to 70%^26^. This pattern may be explained by the biological tropism of HPV for lymphoid-associated structures of the oropharynx, particularly the palatine and lingual tonsils. In these regions, the reticulated epithelium of the tonsillar crypts presents structural discontinuities that facilitate viral access to basal keratinocyte progenitor cells, which are particularly susceptible to HPV-driven carcinogenesis^27,28^. Furthermore, HPV+ tumors were more frequently associated with nodal involvement than HPV− tumors, suggesting differences in clinical presentation according to HPV status, possibly related to the lymphoid-rich tissue commonly involved in HPV-related oropharyngeal tumors^12^.

### Second primary tumor pattern of human papillomavirus-positive and -negative oropharyngeal squamous cell carcinoma patients

According to Martel et al. (2017)^22^, the prevalence of SPTs is higher in HPV– tumors than in HPV+ tumors^14^. In our study, the prevalence of SPTs was 21.8% in HPV– patients compared with 13.0% in HPV+ patients. The most frequent SPT sites in HPV– OPSCC were the head and neck and the respiratory tract, a distribution consistent with the concept of field cancerization, in which the upper aerodigestive epithelium exposed to chronic carcinogenic agents becomes more susceptible to multiple premalignant and malignant lesions^29^. León et al. (2009)^30^ reported an increased risk of SPT among HNC patients who continued smoking (OR 2.9) or consuming alcohol (OR 5.2)^30^. In our cohort, HPV+ OPSCC patients were less strongly associated with smoking and alcohol consumption and have been reported to have a lower risk of SPTs, particularly in anatomical sites related to tobacco and alcohol exposure. In the municipality of São Paulo, the prevalence of smoking among adults aged 18 years or older is 10.3%, the seventh highest among Brazilian capitals, while the prevalence of electronic cigarette use is 3.4%, the second highest in the country^31^. These exposure patterns may help explain the distribution of SPT sites observed in our study. Overall, our findings are consistent with the most frequent SPT locations previously described in HNC^16,32^.

Previous studies have shown that the risk of SPTs remains elevated for several years after the diagnosis of an initial oropharyngeal tumor, particularly for sites associated with tobacco and alcohol exposure, such as the head and neck, esophagus, and lung. Increased standardized incidence ratios have been observed especially during the first years following diagnosis, although the risk persists long-term^33^. In our study, the interval between OPSCC diagnosis and SPT diagnosis was shorter in the HPV+ group than in the HPV– group, indicating that SPTs may occur relatively early during follow-up among HPV+ patients. One possible explanation is that HPV+ tumors are more radiosensitive and are frequently treated with radiotherapy^34^, which may influence the detection or occurrence of subsequent primary tumors over time.

### Public health implications

These findings have important implications for clinical practice and public health strategies. By identifying distinct sociodemographic and clinical patterns in our Brazilian cohort, healthcare providers may improve risk stratification and follow-up planning according to HPV status. HPV+ OPSCC was more frequently observed among individuals without a history of smoking or alcohol consumption, indicating that the absence of classical risk factors should not reduce clinical suspicion for OPSCC. As the incidence of HPV-related oropharyngeal cancer continues to increase independently of tobacco exposure^35^, HPV vaccination may represent an important strategy for HPV-related cancer prevention. Although currently vaccinated cohorts are substantially younger than the populations at highest risk for OPSCC^13^, strengthening vaccination coverage may contribute to long-term reductions in HPV-related oropharyngeal cancer incidence.

SPT remain an important cause of morbidity and mortality among HNC survivors^36^. The occurrence of SPTs in the upper aerodigestive tract highlights the influence of previous tobacco and alcohol exposure on the development of additional primary tumors. Continued exposure to these risk factors after the first diagnosis may further increase the risk of SPTs^33^, including lung cancer, which is associated with poorer survival outcomes. Therefore, promoting smoking and alcohol cessation remains an important component of survivorship care, particularly among HPV- patients.

In Brazil, the organization of oral health services within the Unified Health System (SUS), particularly through the National Oral Health Policy (Política Nacional de Saúde Bucal — PNSB), emphasizes epidemiological surveillance and monitoring of oral diseases of public health relevance, including HNC, supporting cancer prevention, early detection, and coordinated follow-up actions within primary care^37^. Therefore, a team-based approach that includes oral health professionals in the care of HNC patients appears appropriate not only before and during cancer treatment, as currently recommended, but also for early detection and in the post-treatment period^38-40^. In this context, follow-up of patients with OPSCC should consider not only treatment response and adverse effects, as well as the early detection of SPTs, which may improve prognosis and enable timely therapeutic intervention. Differences observed in the occurrence and timing of SPTs between HPV+ and HPV− patients suggest that surveillance strategies may benefit from considering HPV status. A more individualized follow-up approach may contribute to earlier identification of SPTs and improved long-term outcomes, reinforcing the importance of coordinated care pathways between primary care, oral health, and oncology services to reduce the burden of disease.

### Limitations and strengths

There are some limitations that should be considered. First, this is a cohort study conducted in a single tertiary cancer center in Brazil, which may limit the generalizability of the findings, as the study population does not fully represent the broader Brazilian population. In this context, the results should be interpreted with caution and highlight the need to strengthen integrated national health information systems to enable population-based monitoring of OPC and other HPV-related diseases, particularly considering that HPV tumor status is not routinely available in most public databases. Additionally, the retrospective design may have affected the accuracy of some variables, particularly behavioral information such as tobacco and alcohol consumption, which were obtained from medical records and may be subject to misclassification or incomplete reporting. In addition, missing data for some variables may have influenced the multivariable analyses. HPV status was determined using p16 IHC, which is widely used as a surrogate marker; however, discrepancies between p16 IHC and HPV DNA detection have been reported in the classification of HPV-related tumors^41^. Despite these limitations, the availability of detailed clinical information allowed the evaluation of HPV status and SPT patterns in a well-characterized cohort of OPSCC patients. These findings may serve as a reference for future population-based studies and may also contribute to informing strategies for early detection and surveillance of OPSCC and second primary tumors according to HPV status.

In conclusion, the pattern of SPTs in OPSCC differed according to HPV status. HPV− patients showed a higher prevalence of SPTs, more frequently involving the head and neck and respiratory tract, which may be consistent with the cumulative effects of tobacco and alcohol exposure. In contrast, HPV+ patients presented a lower prevalence of SPTs and a distinct distribution of tumor sites, possibly reflecting differences in carcinogenic pathways. The shorter interval between OPSCC and SPT diagnosis observed in HPV+ patients further suggests differences in the timing of SPT occurrence according to HPV status. Together, these findings indicate that HPV+ and HPV- OPSCC present distinct clinical patterns, which may be relevant for risk stratification and follow-up strategies.
