# Supplementary material for: Clinical profile and pattern of second primary tumors in HPV-positive and HPV-negative oropharyngeal cancer
Source: Rev Bras Epidemiol. 2026 Aug 17;29:e260046. doi: 10.1590/1980-549720260046 (PMC13480623; doi:10.1590/1980-549720260046)
Supplement: Supplementary file 1 [file 1980-5497-rbepid-29-e260046-Suppl01.pdf]

Table 1. Sociodemographic and clinical characteristics of OPSCC patients without p16 information. A.C.Camargo Cancer Center (2000–2022).

**Gender**		
Male	630 (83.6)	
Female	124 (16.4)	
**Age (years)**		
< 50	152 (20.2)	
50 - 69	467 (61.9)	
> 69	135 (17.9)	
**Ethnicity**		
White	515 (81.5)	
Non-white	117 (18.5)	
Missig		122
**Education (years)**		
< 10	93 (25.8)	
10 - 12	155 (43.1)	
> 12	112 (31.1)	
Missing		394
**Smoking**		
Smoker	245 (42.1)	
Former smoker	236 (40.5)	
Non smoker	101 (17.4)	
Missing		172
**Alcohol consumption**		
Drinker	257 (46.3)	

Former drinker	164 (29.5)	
Non drinker	134 (24.1)	
Missing		199
**Tumor topography**		
Lymphoepithelial	497 (65.9)	
Non Lymphoepithelial	257 (34.1)	
**Clinical staging**		
I/II	43 (6.6)	
III	143 (21.9)	
IV	466 (71.5)	
Missing		102
**cT (TNM)**		
cT1/cT2	212 (31.2)	
cT3/cT4	467 (68.8)	
Missing		75
**cN (TNM)**		
cN0	219 (32.3)	
cN+	459 (67.7)	
Missing		76
**cM (TNM)**		
cM0	606 (87.4)	
cM1	87 (12.6)	
Missing		61
**Second primary tumor**		
Yes	624 (82.8)	

No	130 (17.2)

Table 2. Topography of second primary tumors in patients with OPSCC stratified by HPV status. A.C.Camargo Cancer Center (2000–2022).

Second primary tumor	HPV+ n=52	HPV- n=61	All n=113
Lips and oral cavity	5 (9.6%)	9 (14.8%)	14 (12.4%)
Oropharynx	1 (1.9%)	4 (6.6%)	5 (4.4%)
Nasopharynx	0 (0.0%)	1 (1.6%)	1 (0.9%)
Hypopharynx	1 (1.9%)	8 (13.1%)	9 (8.0%)
Larynx	1 (1.9%)	1 (1.6%)	2 (1.8%)
Esophagus	1 (1.9%)	4 (6.6%)	5 (4.4%)
Colon and rectum	2 (3.8%)	4 (6.6%)	6 (5.3%)
Digestive tract	1 (1.9%)	2 (3.3%)	3 (2.7%)
Respiratory tract	8 (15.4%)	11 (18.0%)	20 (17.7%)
Skin	9 (17.3%)	2 (3.3%)	11 (9.7%)
Breast	4 (7.7%)	3 (4.9%)	7 (6.2%)
Penis/Prostate	8 (15.4%)	8 (13.1%)	15 (13.3%)
Urinary tract	4 (7.7%)	1 (1.6%)	5 (4.4%)
Nervous system	1 (1.9%)	1 (1.6%)	2 (1.8%)
Thyroid	3 (5.8%)	1 (1.6%)	4 (3.5%)
Other locations	3 (5.8%)	1 (1.6%)	4 (3.5%)

[1]Lips and oral cavity: (C00; C02; C03; C04; C06); Oropharynx: (C05; C09); Nasopharynx: (C11); Hypopharynx: (C12; C13); Larynx: (C10; C32); Esophagus (C15); Colon and rectum (C18; C19; C20); Digestive tract: Stomach; liver, pancreas and digestive tract (C16; C22; C25; C26); Respiratory tract: lungs, trachea and thymus (C33; C34; C37); Skin (C43; C44);); Breast (C50); Uterus and vulva (C51; C54);

Penis/Prostate (C60; C61); Urinary tract: bladder and kidneys (C64; C67); Nervous system: brain and conjunctiva (C69; C72); Thyroid (C73); Other locations: maxillary sinus, retroperitoneum, soft tissue, bone marrow, Hodgkin's lymphoma, non-Hodgkin's lymphoma, peripheral T-cell lymphoma; Other locations NOS (C31; C48; C49; C76; C79; C81; C84; C85)
